# Timely Application of Four Insecticides to Control Corn Earworm and Fall Armyworm Larvae in Sweet Corn

**DOI:** 10.3390/insects13030278

**Published:** 2022-03-11

**Authors:** Diego M. Viteri, Angela M. Linares-Ramírez

**Affiliations:** 1 Department of Agro-Environmental Sciences, University of Puerto Rico, Isabela Research Substation, 2090 Ave. Militar, Isabela, PR 00662, USA; 2 Department of Agro-Environmental Sciences, University of Puerto Rico, Lajas Research Substation, Carr. 101 km 8.5 Barrio Palmarejo, Lajas, PR 00667, USA; angela.linares@upr.edu

**Keywords:** damage on kernels, insecticides, larvae, percentage of mortality, weight

## Abstract

**Simple Summary:**

Corn earworm and fall armyworm are the most important Lepidopteran pests in sweet corn worldwide. Timely application of insecticides during pollination is crucial to control both species and produce kernels with higher quality. Our results showed that applications of insecticides should be conducted at 48 h after pollination. Emamectin benzoate and chlorantraniliprole provided an adequate control of fall armyworm and corn earworm larvae. Furthermore, emamectin benzoate should be applied to larvae in later instars (fourth–sixth) while chlorantraniliprole and spinetoram should be sprayed when infestations of larvae in earlier instars (first–third) are observed.

**Abstract:**

Insecticide sprays are a common practice to control corn earworm, *Helicoverpa zea* (Boddie), and fall armyworm, *Spodoptera frugiperda* (J. E. Smith) (Lepidoptera: Noctuidae), in corn (*Zea mays* L.) at reproductive stages. Our objectives were to determine (1) the most appropriate time for insecticide applications and (2) the effect of four insecticides on the survival of larvae as well as their weight. ß-cyfluthrin (0.4 mL/L), chlorantraniliprole (0.6 mL/L), emamectin benzoate (0.2 g/L), and spinetoram (1.5 mL/L) were sprayed on silks of sweet corn planted in Isabela and Lajas, Puerto Rico 3 h before and 24 and 48 h after pollination. The number of kernels produced and the damage of larvae on kernels were quantified at harvest. In addition, percentages of mortality and changes on larval weight were noted at 96 h after insecticide applications. Insecticide sprays at 3 h before pollination reduced the number of kernels or were similar to the control in all treatments. However, emamectin benzoate sprayed in Lajas and chlorantraniliprole applied in Isabela at 48 h after pollination increased the number of kernels (281–294) and reduced the damage of larvae on kernels (<0.5%) compared to the control (201–229; >7%). Furthermore, applications of emamectin benzoate caused higher percentages of fall armyworm larval mortality (>70%). Conversely, ß-cyfluthrin and chlorantraniliprole caused lower percentages of mortality (<30%) and only chlorantraniliprole and spinetoram reduced the weight of corn earworm and fall armyworm larvae collected in both locations. This information may help pest management programs and corn breeders to schedule insecticide sprays and pollination in the field.

## 1. Introduction

Corn earworm, *Helicoverpa zea* (Boddie) (Lepidoptera: Noctuidae), and fall armyworm, *Spodoptera frugiperda* (J. E. Smith) (Lepidoptera: Noctuidae), are the most important pests affecting field/sweet corn (*Zea mays* L.) in the tropics, the United States, and worldwide [1,2,3,4,5,6]. Larvae of fall armyworm feed on leaves, tassels, and kernels [7] while corn earworm larvae are mostly observed feeding on kernels or cob tissue at reproductive stages [4]. Later larval instars (fourth to sixth) cause higher damage in the plant tissue, and yield losses over 18% have been reported for both species [7,8,9]. 

Puerto Rico is an important winter nursery location mostly for field corn with more than 1000 ha planted in each season. In fact, private companies and universities from North America use Puerto Rico to advance generations, and for genetics and breeding studies [10]. The tropical conditions of Puerto Rico, multiple plantings, and the presence of other hosts (e.g., soybean, rice, and vegetables) allow the development of multiple generations (>10) of both pest species during the year [11]. Furthermore, resistance of corn earworm and fall armyworm larvae to different insecticides including *Bacillus thuringiensis*, carbamates, and pyrethroids [2,10,12,13,14] were reported. All of these factors have caused biological, chemical, cultural, and mechanical controls plus fallow periods to be necessary to combat and decrease the populations of both species in each season. 

Chemical control in combination with biological insecticides is the most common strategy to control Lepidopteran species in Puerto Rico [10,11]. In fact, some insecticides (e.g., ß-cyfluthrin and spinetoram) have been used widely in direct applications to cobs after pollination to avoid the damage of corn earworm and fall armyworm larvae and other pests (e.g., corn sap beetle, *Carpophilus* spp. (Coleoptera: Nitidulidae); and silk fly *Euxesta* spp. (Diptera: Ulidiidae)) in the kernels (Viteri D, personal communication). Likewise, the efficacy of chlorantraniliprole, methoxyfenozide, and spinosyn to control corn earworm and fall armyworm larvae at reproductive stages by the reduction of the damage in the ears/kernels of sweet corn were reported in experimental trials in Florida and New York [15,16,17]. However, there are no previous studies, to the best of our knowledge, related to the appropriate time when the insecticides need to be sprayed (before or after pollination) to avoid any damage in the fertilization and kernel yields. 

The intensive insecticide sprays (i.e., over 20 applications per season [2]) and the higher larvae pressure of both species in field/sweet corn in Puerto Rico have been important issues for pest management programs who want to avoid or delay the resistance to insecticides, and corn breeders who need to obtain higher numbers of viable seeds in their crosses or seed increases. In fact, an adequate synchronization at the time of pollination and insecticide sprays are necessary to meet the desired production of kernels. For this, insecticide rotations with different modes of action and short reentry periods (≤12 h) (e.g., chlorantraniliprole, emamectin benzoate, and spinetoram) should be used at the peak of pollinations. The objectives of this study were to determine (1) the most appropriate time for insecticide applications and (2) the effect of four insecticides on corn earworm and fall armyworm larval survival, as well as their weight.

## 2. Materials and Methods

### 2.1. Field Experiments

Eight experimental trials with sweet corn ‘Suresweet 2011’ were planted in Isabela and Lajas Research Substations at the University of Puerto Rico in September 2020 for emamectin benzoate treatment, and February, March, and April 2021 for spinetoram, chlorantraniliprole, and ß-cyfluthrin treatments, respectively. The Isabela substation is located at latitude 18°30′00″ N, longitude 67°00′00″ W, and at 126 m above sea level. The temperature varies from 20 to 31 °C with a mean annual precipitation of 1592 mm, and 70% relative humidity. The soil at the Isabela Substation belongs to the Oxisol type (Coto series) [18]. The Lajas substation is located at 18°03′ 00″ N, 67°03′34″ W at 9 m above the sea level. Lajas has soils belonging to the Mollisol and Vertisol orders, and has temperatures between 19 to 33 °C, an annual precipitation of 1143 mm, and an average relative humidity of 80% [18].

Experimental plots were 25 × 15 m in size. One sweet corn seed was planted every 0.30 m in each row (25 m length) and the separation between rows was 0.76 m. Each treatment was placed in every 4 rows. Plants with cobs at R1 stage, without visible silk hairs, were covered with shoot bags to avoid their exposure to the pollen. Then, 5 mL of each insecticide solution was sprayed at higher dosages (Table 1) to four-day-old silk hairs, 3 h before pollination and 24 and 48 h after pollinations by the syringe Ideal^®^ Prima^®^ backpack (Neogen, Lansing, MI, USA). A total volume of 150 mL of insecticide solution was used per treatment at each time of pollination. The control was not sprayed. Sixty milligrams of fresh pollen (50–55% of moisture content) per silk was used for pollinations and tassel bags replaced the shoot bags when plants were pollinated. A randomized complete block design with three replications was used in each location, and ten cobs were used per each insecticide treatment and the control per replication (*n* = 120 per location). Cobs were harvested 21 days after the pollination. The number of kernels produced and the percentage of corn earworm and fall armyworm damage on the kernels under natural infestation were recorded. 

### 2.2. Bioassays

Larvae were collected from leaves and ears of sweet corn ‘Suresweet 2011’ planted in Isabela and Lajas Research Substations at the University of Puerto Rico in June 2019 for corn earworm and June 2020 for fall armyworm. The weight of each larva was recorded, and then one fourth or fifth instar larva of corn earworm or fall armyworm was placed separately in a 20 mL plastic cup (Lion, Santo Domingo, Dominican Republic) containing soybean–wheat germ-based artificial diet (Frontier Scientific Services Inc., Newark, DE, USA). Fifteen larvae of each species per replication were treated topically with 3 mL (200 μL per larva) insecticide solution of ß-cyfluthrin, chlorantraniliprole, emamectin benzoate, and spinetoram per replication at the dosages described in Table 1. A micropipette with a 200 μL Eppendorf tip was used to deliver the insecticide solution over the head, thorax, and abdomen of each larva. The control was sprayed with 200 μL of distilled water per larva. Treated cups and the control were held in a randomized complete block design with 4 replications (total *n* = 60 per location and species) in the laboratory at 18 to 20 °C, and a photoperiod of 12:12 h (L:D). The percentage of mortality and the weight of each larva were evaluated at 96 h after the insecticide application. 

### 2.3. Data Analysis

A combined data analysis [19] was performed to determine the effect of the location and the interactions location x time for pollination and location x larval weight per each insecticide. A fixed model was used for data analysis where the two locations, time of pollination, and larval weight were fixed effects and the replication was a random effect [19]. In addition, simple analysis of variance for the number of kernels produced, the percentage of corn earworm and fall armyworm damage on kernels, and the larval weight were conducted in each location. Similar criterion was used for the percentage of larval mortality in the bioassays. However, the four insecticide treatments (also a fixed effect) were analyzed together in the combined and simple analysis. Data were analyzed using SAS 9.4 PROC GLIMMIX [20] and a TUKEY HRS test was conducted to discriminate differences among treatments versus the control for each variable. In addition, Abbott’s formula [21] was used to correct larval mortality attributed to the insecticide effect in the bioassays. 

## 3. Results

Significant differences (*p* ≤ 0.05) between the time of application were observed for all insecticides in the number of kernels produced (Table 2). The interaction location x time of application showed significant differences for ß-cyfluthrin, chlorantraniliprole, and spinetoram for the same variable. On the contrary, significant differences for the time of application and its interaction with the location were only observed for chlorantraniliprole and emamectin benzoate for the variable damage on kernels (Table 2). The simple analysis of variance also showed differences between time of application for all insecticide treatments in the number of kernels produced in both locations (Table 3). Emamectin benzoate showed differences for the time of application in Isabela and Lajas for the damage on kernels (Table 3).

The combined analysis for the percentage of mortality had significant differences between the location and location x treatment interaction for corn earworm and fall armyworm (Table 4). Similar results were observed for the simple analysis of variance for corn earworm (*F* = 6.68; df = 4, 12; *p* ≤ 0.005 and *F* = 79.47; df = 4, 12; *p* ≤ 0.0001) and fall armyworm (*F* = 45.64; df = 4, 12; *p* ≤ 0.0001 and *F* = 154.49; df = 4, 12; *p* ≤ 0.0001) in Isabela and Lajas, respectively. In addition, significant differences were observed between larval weight and location x larval weight interaction for ß-cyfluthrin for corn earworm (Table 5). Conversely, larval weight and location x larval weight showed significant differences for the control, chlorantraniliprole, and spinetoram treatments for fall armyworm (Table 5). Furthermore, significant differences were noted for chlorantraniliprole and spinetoram in the simple analysis of variance carried out for both species in Isabela and Lajas (Table 6). 

In general, insecticide applications at 3 h before pollination or 24 h after pollination reduced the number of kernels produced or were similar to the control, especially in lower corn earworm and fall armyworm infestations (Table 7). In contrast, ß-cyfluthrin and emamectin benzoate sprayed at 48 h after pollination increased the number of kernels (means of 268 and 281, respectively). However, only the applications of emamectin benzoate reduced the damage of corn earworm and fall armyworm larvae in the kernels (<0.5%) compared to the control (229; 7.3%) in Lajas. Likewise, applications of chlorantraniliprole at the same time increased the yield (294) and produced kernels without damage compared to the control (201; 9%) in Isabela.

The applications of emamectin benzoate caused 80 and 94% of larval mortality from fall armyworm populations collected in Isabela and Lajas, respectively. However, corn earworm larvae collected in Isabela had lower percentages of mortality (36%) to this insecticide. Spinetoram caused between 53 to 70% of larval mortality in Lajas and 30 to 36% in Isabela for both species. Conversely, applications of ß-cyfluthrin and chlorantraniliprole caused percentages of mortality below 27% in both populations (Table 8 and Table 9). Corn earworm and fall armyworm larvae treated with chlorantraniliprole and spinetoram had a significant weight reduction in both locations (Table 8 and Table 9). In contrast, there were not significant differences in the weight in larvae of both populations applied with ß-cyfluthrin and emamectin benzoate, while corn earworm and fall armyworm larvae treated with water had similar or higher weight at 96 h after insecticide application in both locations as expected (Table 8 and Table 9). 

## 4. Discussion

Our data suggest that safe insecticide sprays on silks should be conducted at 48 h or longer after pollination. Applications of commercial dosages of ß-cyfluthrin and chlorantraniliprole at 3 h before pollination and 24 h after pollination reduced the number of kernels produced > 20% in Isabela. Likewise, applications of emamectin benzoate and spinetoram in Isabela and chlorantraniliprole and spinetoram in Lajas at 3 h before pollination reduced the seed yield from 23 to 60%. Apparently, the water or the insecticide solution applied on the silks may affect the pollen germination, female receptivity or the syngamy process which occurs in the next 28 h after pollination [22,23]. In fact, the extracellular surface of corn silks has lipids with non-polar molecules [24]. This hydrophobic property of the silks caused bubbles of water to form on the surface when insecticides were sprayed (especially at earlier insecticide applications). The residues of water on the silk hairs/trichomes may kill some pollen grains, avoiding proper fertilization. In addition, corn silks provide pollen hydration for its germination when pollen is in contact with the stigma [23], and the water or insecticide solutions may also affect these biological procedures. Further research is necessary to estimate the time required for the water evaporation on the silks or if the insecticide residues in the corn plant tissue cause a detrimental effect on the pollen–pistil interactions. Residues of acephate, acetamiprid, chromafenozide, and etofenprox were reported on silks and husks of sweet corn [25]. Furthermore, the residues of the four insecticides used in this study vary from 5 to 11 days in the plant tissue [26,27,28,29]. However, our data suggest that residues of insecticide solutions on silks up to 24 h may cause a negative effect in the pollen, fertilization, and/or kernel formation. In vitro studies should be carried out with pollen germination media [30] amended with ß-cyfluthrin, chlorantraniliprole, emamectin benzoate, and spinetoram at different dosages to verify if these molecules cause any damage in the pollen. It has been reported that corn pollen germination and tube elongation were inhibited or reduced by the combination of trichlorfon with adjuvants [31]. 

Chlorantraniliprole was the most effective treatment in the field in Isabela at 48 after pollination. Likewise, spinetoram reduced the damage of Lepidopteran species on kernels at the same period although there were not statistical differences compared with the control. The direct application of both insecticides on the corn earworm and fall armyworm larvae caused lower percentages of mortality. However, there was a marked effect in the weight reduction in the larvae treated with these active ingredients. It has been reported that diamides cause an impaired regulation of muscle contraction [32] while spinosyns produce larvae paralysis attributed to fatigue in the nervous system [33]. Future studies are necessary to determine the rate of larvae feeding and their relationship with the damage in kernels from larvae treated with chlorantraniliprole and spinetoram. It is important to mention that larvae in earlier instars (first–third) of corn earworm (80%) and fall armyworm (20%) were observed on silks at the time of applications. Thus, these stages probably were more susceptible to chlorantraniliprole and spinetoram and/or their indirect effect in the weight reduction compared to the larvae in fifth instar that were used in the bioassays.

Emamectin benzoate sprayed at 48 after pollination was effective to control Lepidopteran species in Lajas where the rate of corn earworm and fall armyworm populations in cobs reached up to 1:1, as reported previously [10]. In fact, our data showed that both larvae species treated with emamectin benzoate had higher percentages of mortality in Lajas. On the contrary, corn earworm populations from Isabela did have lower percentages of mortality, and this may be associated with the prolonged use of this pesticide to control corn earworm in other crops (e.g., common bean), compared to Lajas where most of the applications are conducted with biological insecticides. In addition, the percentages of mortality reached 55% when corn earworm larvae in the fourth instar were treated with emamectin benzoate in Isabela (Viteri D, unpublished). Sarmiento et al. (2022) [34] also reported percentages of corn earworm mortality between 50 to 75% of populations from Isabela, Lajas, and Juana Díaz in previous years.

In summary, insecticides should be applied at least 48 h after pollination to control severe infestations of fall armyworm and corn earworm larvae on cobs/kernels of sweet corn. Emamectin benzoate provided an adequate control for both Lepidopteran species. If a second application is required or under infestations of earlier larvae instars (first–third), spinetoram or chlorantraniliprole could be used in rotation. Furthermore, the evaluation of synthetic insecticides in combination with biological control agents [14,35] is necessary in targeted applications on silks and cobs.

## Figures and Tables

**Table 1:** Active ingredients and their percentages, insecticide group, commercial names, manufacturers and dosages of four insecticides used in field applications and bioassays to control corn earworm (*Helicoverpa zea* (Boddie) (Lepidoptera: Noctuidae)) and fall armyworm (*Spodoptera frugiperda* (J. E. Smith) (Lepidoptera: Noctuidae)) larvae in 2019 through 2021.

Insecticides	Major Group or Subgroup	Commercial Name	Manufacturers	Dosage (g or mL/L)
Active ingredient and percentage				
ß-cyfluthrin 12.70%	3 A (pyrethroid)	Baythroid^®^XL	Bayer	0.40 mL
Chlorantraniliprole 18.40%	28 (diamide)	Coragen^®^	Dupont	0.60 mL
Emamectin benzoate 2.12%	6 (avermectin)	Proclaim^®^05SG	Syngenta	0.20 g
Spinetoram 11.70%	5 (spinosyn)	Radiant^®^SC	Dow AgroSciences	1.50 mL

**Table 2:** Combined analysis of variance for the number of kernels produced and percentages of damage of corn earworm (*Helicoverpa zea* (Boddie) (Lepidoptera: Noctuidae)) and fall armyworm (*Spodoptera frugiperda* (J. E. Smith) (Lepidoptera: Noctuidae)) per each insecticide in sweet corn (*Zea mays* L.) ‘Suresweet 2011’ planted at Isabela and Lajas, Puerto Rico in 2020 and 2021.

Effect	df	Number of Kernels Produced				Damage on Kernels			
		ß-cyfluth- rin	Chlorantra-niliprole	Emamectin benzoate	Spineto- ram	ß-cyfluth- rin	Chlorantra-niliprole	Emamectin benzoate	Spineto- ram
		*F* values							
Location (L)	1, 2	43.97 *	5.18	12.71	4.58	3.38	3.49	2.37	6.24
Time of application (T)	3, 228	39.92 ***	27.09 ***	7.18 ***	11.64 ***	0.89	10.15 ***	3.46 *	3.67 *
L × T	3, 228	30.04 ***	9.56 ***	1.08	18.88 ***	2.13	10.15 ***	4.00 *	0.92

* Significant at *p* ≤ 0.05, and *** *p* ≤ 0.001.

**Table 3:** Simple analysis of variance for the number of kernels produced and percentages of damage of corn earworm (*Helicoverpa zea* (Boddie) (Lepidoptera: Noctuidae)) and fall armyworm (*Spodoptera frugiperda* (J. E. Smith) (Lepidoptera: Noctuidae)) per each insecticide in sweet corn (*Zea mays* L.) ‘Suresweet 2011’ planted at Isabela and Lajas, Puerto Rico in 2020 and 2021.

Variable	Effect	df	Isabela				Lajas			
			ß-cyfluth- rin	Chloran-traniliprole	Emamec- tin benzoate	Spineto- ram	ß-cyfluth- rin	Chloran-traniliprole	Emamec- tin benzoate	Spineto- ram
			*F* values							
Number of kernels produced	Time of application	3, 114	100.67 ***	22.04 ***	4.11 *	11.17 ***	10.44 ***	6.62 ***	4.10 *	20.68 ***
Damage on kernels	Time of application	3, 114	3.30 *	10.15 ***	2.83 *	2.12	1.38	_–_^a^	3.81 *	4.23 *

* Significant at *p* ≤ 0.05, and *** *p* ≤ 0.001. ^a^ There was no infestation of corn earworm and fall armyworm larvae. Thus, statistical analyses were not conducted.

**Table 4:** Combined analysis of variance for the percentages of mortality of corn earworm (*Helicoverpa zea* (Boddie) (Lepidoptera: Noctuidae)) and fall armyworm (*Spodoptera frugiperda* (J. E. Smith) (Lepidoptera: Noctuidae)) larvae evaluated at 96 h after insecticide application in 2019 and 2020.

Effect	df	Corn Earworm	Fall Armyworm
		*F* values	
Location (L)	1, 3	20.11 *	9.29
Treatment (T)	4, 24	47.07 ***	157.58 ***
L × T	4, 24	12.69 ***	12.71 ***

* Significant at *p* ≤ 0.05, and *** *p* ≤ 0.001.

**Table 5:** Combined analysis of variance for the location and weight of corn earworm (*Helicoverpa zea* (Boddie) (Lepidoptera: Noctuidae)) and fall armyworm (*Spodoptera frugiperda* (J. E. Smith) (Lepidoptera: Noctuidae)) larvae evaluated at 96 h after insecticide application in 2019 and 2020.

Effect	df	Corn Earworm					Fall Armyworm				
		No insectici- de	ß-cyfluth- rin	Chloran-tranilipro- le	Emame-ctin benzo- ate	Spine- toram	No insetici- de	ß-cyfluth-rin	Chloran-tranilipro- le	Emame- ctin benzo- ate	Spine-toram
		*F* values									
Location (L)	1, 3	6.57	0.74	28.05 *	17.27 *	10.09	169.52 **	27.82 *	190.54 ***	20.08 *	511.86 ***
Larval weight (W)	1, 230	300.47 ***	18.36 ***	15.11 ***	0.01	16.37 ***	7.75 **	2.01	74.40 ***	1.63	81.94 ***
L × W	1, 230	0.08	8.10 **	0.86	0.46	0.10	9.58 **	9.05 **	28.09 ***	0.60	43.81 ***

* Significant at *p* ≤ 0.05, **p ≤ 0.01, and ***p ≤ 0.001.

**Table 6:** Simple analysis of variance for the weight of corn earworm (*Helicoverpa zea* (Boddie) (Lepidoptera: Noctuidae)) and fall armyworm (*Spodoptera frugiperda* (J. E. Smith) (Lepidoptera: Noctuidae)) larvae evaluated at 96 h after insecticide application in 2019 and 2020.

Effect	df	Location	Corn Earworm					Fall Armyworm				
			No insecticide	ß-cyfluthrin	Chlorantra-niliprole	Emamec-tin benzo- ate	Spineto- ram	No insecticide	ß-cyfluthrin	Chlorantra-niliprole	Emamec-tin benzo- ate	Spineto- ram
			*F* values									
Larval weight	1, 115	Isabela	100.44 ***	0.90	3.84 *	0.15	5.13 *	0.04	1.05	63.09 ***	1.65	80.56 ***
	1, 115	Lajas	71.80 ***	30.03 ***	13.40 ***	0.17	14.83 ***	23.45 ***	12.40 ***	11.94 ***	0.17	6.22 **

* Significant at *p* ≤ 0.05, ** *p* ≤ 0.01, and *** *p* ≤ 0.001.

**Table 7:** Mean (±SE) of the number of kernels produced at different time of insecticide applications and percentages of damage of corn earworm (*Helicoverpa zea* (Boddie) (Lepidoptera: Noctuidae)) and fall armyworm (*Spodoptera frugiperda* (J. E. Smith) (Lepidoptera: Noctuidae)) larvae in kernels of sweet corn (*Zea mays* L.) ‘Suresweet 2011′ planted at Isabela and Lajas, Puerto Rico in 2020 and 2021.

		Isabela		Lajas	
Treatment ^+^	Time of Application	Number of Kernels	Damage on Kernels %	Number of Kernels	Damage on Kernels %
					
ß-cyfluthrin	Control (no insecticide)	264 ± 7.06 a	0.80 ± 0.34 a	190 ± 12.09 a	3.60 ± 1.62 a
	3 h before pollination	86 ± 8.68 b	2.10 ± 0.73 b	202 ± 9.54 a	2.20 ± 1.13 a
	24 h after pollination	140 ± 9.91 b	0.50 ± 0.13 a	257 ± 13.09 b	3.20 ± 1.21 a
	48 h after pollination	234 ± 6.84 b	0.70 ± 0.21 a	268 ± 13.55 b	6.30 ± 2.05 a
Chlorantraniliprole	Control (no insecticide)	201 ± 22.90 a	8.60 ± 2.72 a	151 ± 13.83 a	0.00 ± 0.00 a
	3 h before pollination	50 ± 7.85 b	0.10 ± 0.06 b	94 ± 8.37 b	0.00 ± 0.00 a
	24 h after pollination	159 ± 25.57 b	0.00 ± 0.00 b	157 ± 12.28 a	0.00 ± 0.00 a
	48 h after pollination	294 ± 25.27 b	0.00 ± 0.00 b	159 ± 13.02 a	0.00 ± 0.00 a
Emamectin benzoate	Control (no insecticide)	219 ± 12.65 a	0.40 ± 0.14 a	229 ± 17.59 a	7.30 ± 3.21 a
	3 h before pollination	169 ± 14.44 b	1.70 ± 0.95 a	214 ± 12.98 a	1.00 ± 0.54 b
	24 h after pollination	202 ± 12.79 a	0.00 ± 0.00 a	228 ± 13.38 a	1.80 ± 0.61 b
	48 h after pollination	226 ± 9.25 a	0.00 ± 0.00 a	281 ± 13.78 b	0.40 ± 0.19 b
Spinetoram	Control (no insecticide)	197 ± 23.12 a	8.78 ± 2.47 a	232 ± 10.98 a	0.91 ± 0.65 a
	3 h before pollination	75 ± 22.96 b	2.82 ± 2.18 a	93 ± 10.62 b	0.30 ± 0.27 a
	24 h after pollination	199 ± 12.12 a	4.50 ± 5.65 a	211 ± 14.41 a	0.00 ± 0.00 b
	48 h after pollination	217 ± 17.68 b	2.52 ± 1.32 a	224 ± 15.34 a	0.00 ± 0.00 b

**^+^** TUKEY HRS test was conducted separately for each insecticide per location. Different letters denote significant differences at *p* ≤ 0.05 for the number of kernels produced and damage on kernels between the time of each insecticide application versus the control.

**Table 8:** Mean (±SE) of percentages of mortality for corn earworm (*Helicoverpa zea* (Boddie) (Lepidoptera: Noctuidae)) larvae and their initial and final weight evaluated at 96 h after insecticide application in 2019.

Treatment	Isabela			Lajas		
	% of Mortality ^+^	Initial Weight (g)	Final Weight (g)	% of Mortality	Initial Weight (g)	Final Weight (g)
Control	0.00 ± 0.00 a	0.22 ± 0.01 a	0.42 ± 0.02 b	0.00 ± 0.00 a	0.18 ± 0.01 a	0.38 ± 0.01 b
ß-cyfluthrin	26.52 ± 8.08 b	0.25 ± 0.01 a	0.27 ± 0.02 a	21.33 ± 6.01 b	0.18 ± 0.01 a	0.30 ± 0.02 b
Chlorantraniliprole	10.24 ± 6.43 a	0.22 ± 0.01 a	0.18 ± 0.01 b	16.67 ± 5.89 b	0.17 ± 0.01 a	0.12 ± 0.01 b
Emamectin benzoate	32.74 ± 7.68 b	0.28 ± 0.02 a	0.27 ± 0.02 a	91.90 ± 3.00 b	0.20 ± 0.01 a	0.21 ± 0.01 a
Spinetoram	35.92 ± 3.42 b	0.21 ± 0.01 a	0.17 ± 0.01 b	53.77 ± 3.70 b	0.18 ± 0.01 a	0.13 ± 0.01 b

^+^ Different letters denote significant differences at *p* ≤ 0.05 between the percentages of mortality of larvae treated with each insecticide versus the control according to the TUKEY HRS test. Different letters denote significant differences at *p* ≤ 0.05 between the initial and the final weight according to the TUKEY HRS test.

**Table 9:** Mean (±SE) of percentages of mortality for fall armyworm (*Spodoptera frugiperda* (J. E. Smith) (Lepidoptera: Noctuidae)) larvae and their initial and final weight evaluated at 96 h after insecticide application in 2020.

Treatment		Isabela		Lajas		
	% of Mortality ^+^	Initial Weight (g)	Final Weight (g)	% of Mortality	Initial Weight (g)	Final Weight (g)
Control	0.00 ± 0.00 a	0.29 ± 0.01 a	0.29 ± 0.02 a	0.00 ± 0.00 a	0.09 ± 0.01 a	0.16 ± 0.01 b
ß-cyfluthrin	8.70 ± 4.15 a	0.26 ± 0.01 a	0.24 ± 0.02 a	5.77 ± 3.68 a	0.12 ± 0.01 a	0.18 ± 0.02 b
Chlorantraniliprole	22.14 ± 7.51 b	0.30 ± 0.01 a	0.20 ± 0.06 b	9.62 ± 5.77 a	0.11 ± 0.01 a	0.08 ± 0.005 b
Emamectin benzoate	79.80 ± 4.31 b	0.22 ± 0.01 a	0.20 ± 0.01 a	94.24 ± 3.68 b	0.10 ± 0.01 a	0.09 ± 0.01 a
Spinetoram	30.00 ± 3.74 b	0.29 ± 0.01 a	0.18 ± 0.01 b	70.36 ± 0.36 b	0.09 ± 0.004 a	0.07 ± 0.01 b

^+^ Different letters denote significant differences at *p* ≤ 0.05 between the percentages of mortality of larvae treated with each insecticide versus the control according to the TUKEY HRS test. Different letters denote significant differences at *p* ≤ 0.05 between the initial and the final weight according to the TUKEY HRS test.

## Data Availability

Not applicable.
